# Supplementary material for: Transcriptional repression of Plxnc1 by Lmx1a and Lmx1b directs topographic dopaminergic circuit formation
Source: Nat Commun. 2017 Oct 16;8:933. doi: 10.1038/s41467-017-01042-0 (PMC5643336; doi:10.1038/s41467-017-01042-0)
Supplement: Supplementary file 1 — Supplementary Information [file 41467_2017_1042_MOESM1_ESM.pdf]

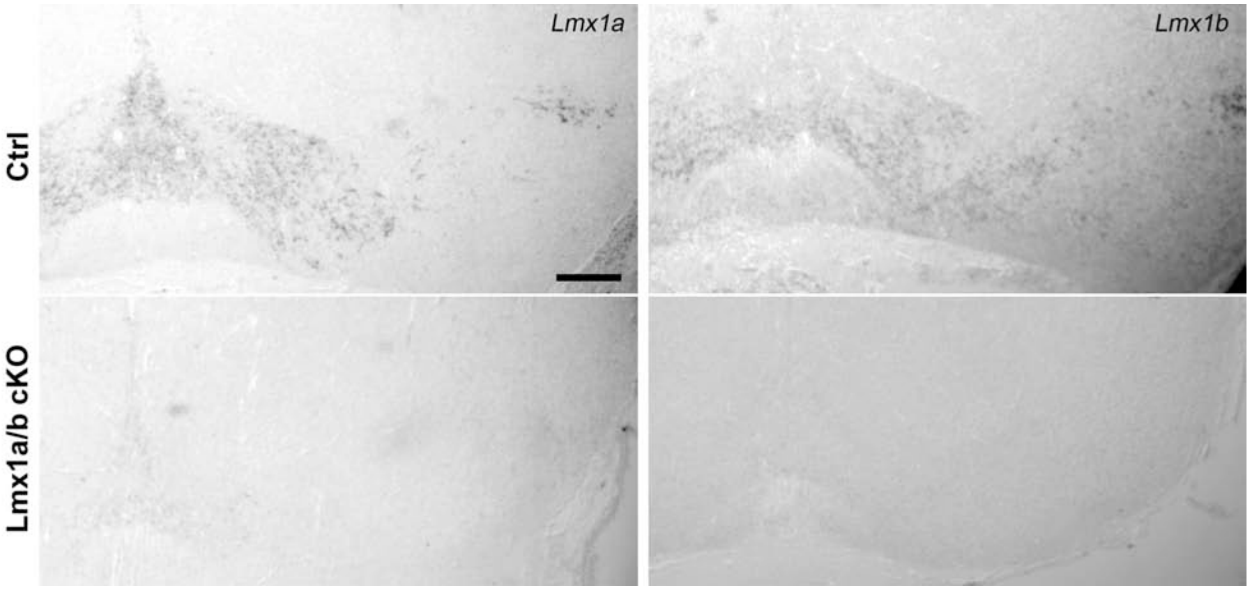

Supplementary Figure 1: Lmx1a and Lmx1b are deleted in mDA neurons of Lmx1a/b cKO mice. *In situ* hybridization for Lmx1a and Lmx1b on Lmx1a/b cKO and control midbrain sections at P1 showing the absence of Lmx1a and Lmx1b expression in the Lmx1a/b cKO midbrain. Scale bar: 250 μm.

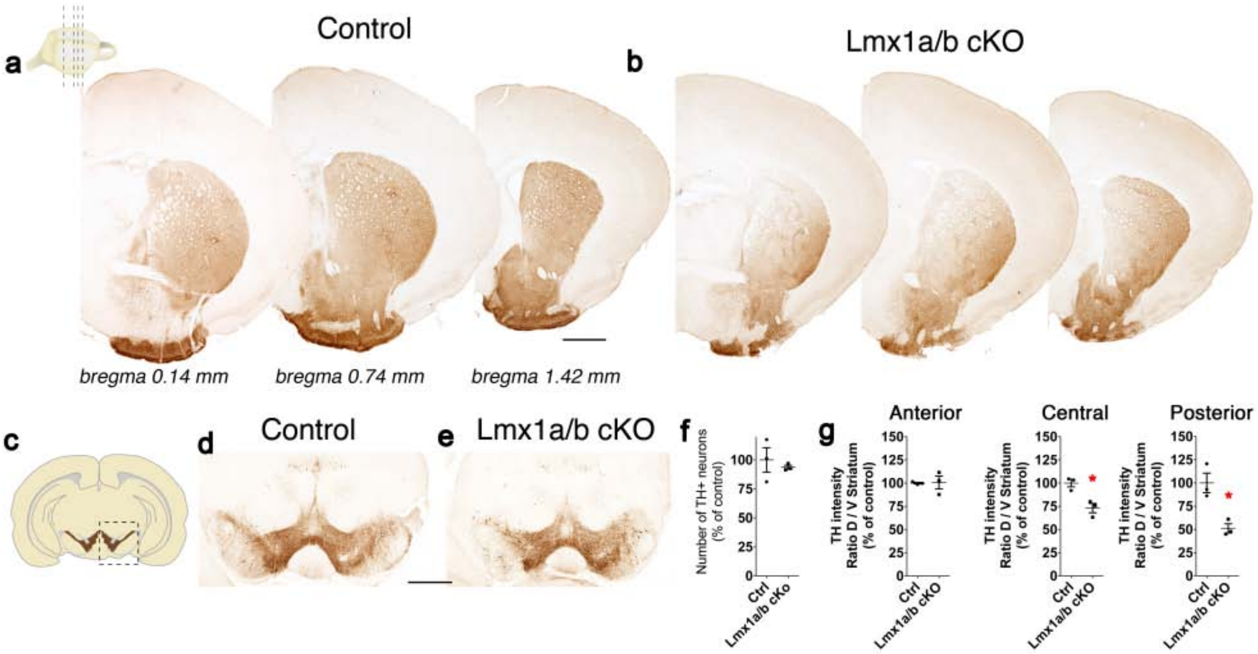

Supplementary Figure 2: Phenotype analysis of Lmx1a/cKO mutant mice at P15. (**a** and **b**) Representative images of TH immunostaining of coronal striatal sections at P15 in control (**a**) and double conditional mutant mice for Lmx1a/b (**b**) showing a loss of innervation in the dorsal striatum for the mutant. (**c-e**) Schematic and representative images of TH immunostaining of coronal midbrain sections for the control and Lmx1a/b cKO mutant showing no change in the number of mDA cells as quantified by stereological counting of TH-positive cells in (**f**) (n=3, Mann-Whitney U, p = 0.700). (**g**) Optical density measurements of TH axons in the striatum. Graphs show the ratio of TH intensity in dorsal *vs* ventral striatum (n=3, Mann-Whitney U, $p^{(Ant\ D/V)}$ = 0.7000, two-tailed unpaired t-test, $p^{(Cent\ D/V)}$ = 0.0140, $p^{(Post\ D/V)}$ = 0.0138). Scale bar: 250 μm.

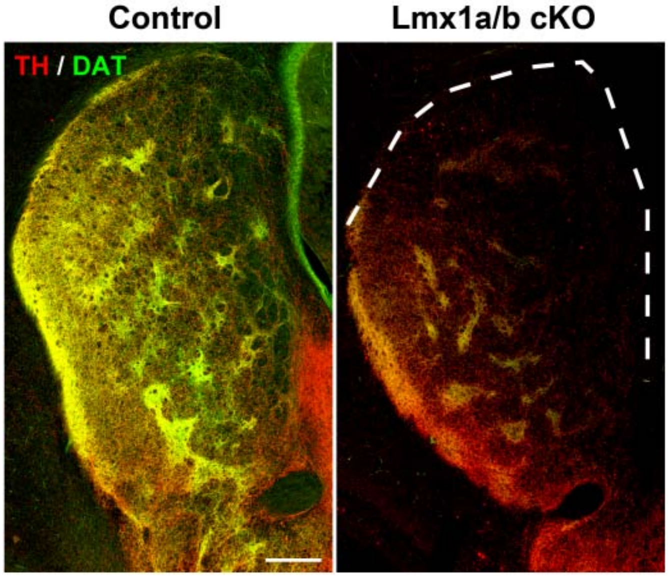

Supplementary Figure 3: Lack of TH and DAT expression in Lmx1a/cKO mutant mice. Confocal images of the dopaminergic markers TH (in red) and DAT (in green) in the striatum of control and Lmx1a/b cKO mutant were performed to show the loss of dopaminergic innervation using these two specific dopaminergic markers. Scale bar: 200 μm.

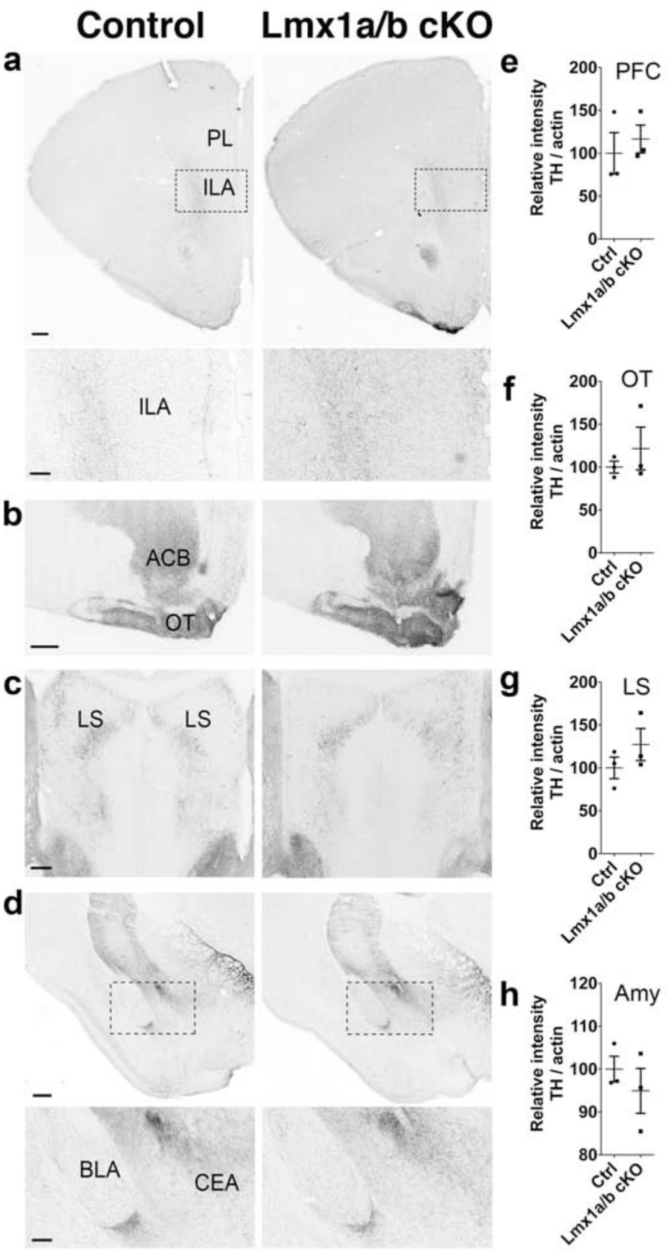

Supplementary Figure 4: Extrastriatal targets in Lmx1a/b cKO mutant mice and control at P15. (a-d) Representative images of TH-stained coronal sections in the extrastriatal dopaminergic targets do not show abnormal DA innervation in Lmx1a/b cKO mice as shown in (a) at the PFC level; (b) Olfactory tubercule; (c) Septum; (d) Amygdale. (**e-h**) Optical density measurement of extrastriatal targets at P15 comparing controls and Lmx1a/b cKO (n=3, two-tailed unpaired t-test: $p^{(PFC)} = 0.5986$, $p^{(OT)} = 0.4489$, $p^{(LS)} = 0.2931$, $p^{(Amy)} = 0.4472$). Optical density measurements in the prefrontal cortex were performed at 3 different antero-posterior levels in prelimbic and infralimbic areas (Bregma +2.22 mm, +1.98 mm and +1.70 mm). Dashed boxes in a and d delineate the higher magnification images in the lower panels. The optical density measurements in the septum were performed at 3 different antero-posterior levels (Bregma +1.18mm, +0.62mm; and +0.14mm) in the area located between the 2 lateral ventricles and between the corpus callosum (upper border) and the zona limitans (lower border). The optical density measurements in the olfactory tubercule were performed at 3 different antero-posterior levels (Bregma +1.73mm, +1.10mm and +0.62mm). For the Amygdala, measurements were performed in the central amygdaloid nucleus (Bregma -1.80mm). Abbreviations: Prelimbic area of the prefrontal cortex (PL), Infralimbic area of the prefrontal cortex (ILA), prefrontal cortex (PFC), Nucleus accumbens (ACS), olfactory tubercule (OT), lateral septum (LS), Basolateral amygdalar nucleus (BLA), Central amygdalar nucleus (CEA) and amygdale (Amy). Scale bars= 250 μm.

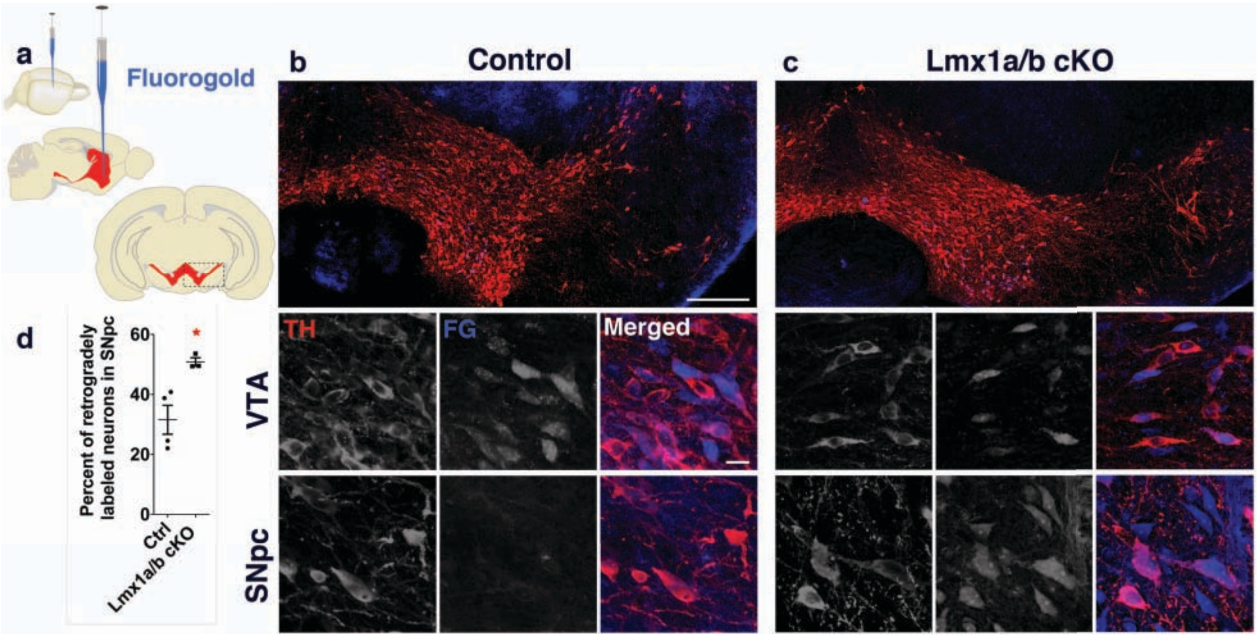

Supplementary Figure 5: Retrograde tracing experiment showing aberrant DA connections in Lmx1a/b cKO. (**a**) Schematic representation of the injection site of the Fluorogold retrograde tracer in the ventral striatum. (**b-c**) Representative confocal images of the retrogradely labeled cells in control (**b**) (TH in red, Fluorogold in blue) and in Lmx1a/b cKO mutant mice (**c**). Lower panels show higher magnification in the VTA and SNpc. (**d**) Quantification of the percentage of retrogradely labeled neurons in SNpc (GFP+TH+ in SNpc on total GFP+TH+; n=4 for controls and n=3 for Lmx1a/b cKO mice; two-tailed unpaired t-test, p = 0.0208). Scale bars: b upper panel 250 µm, lower panels, 15 µm.

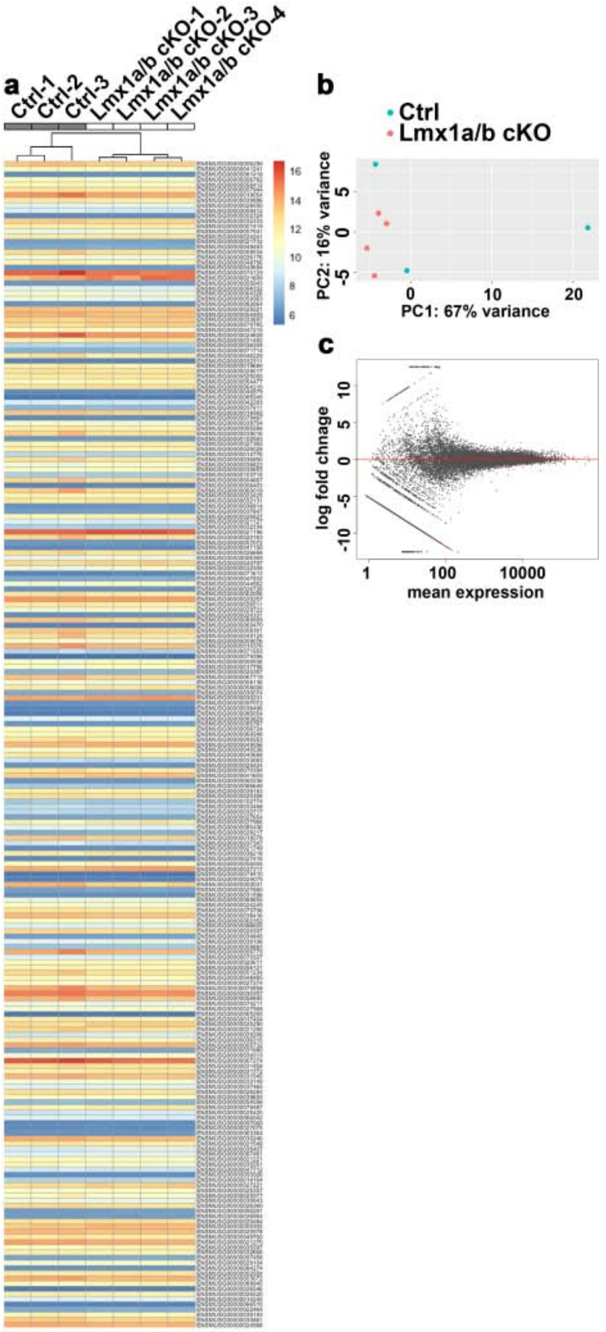

Supplementary Figure 6: Gene expression profiling of control and Lmx1a/b cKO at E15.5. a) A clustered heatmap showing transformed expression values of 225 differentially expressed genes, with adjusted p value of 0.2. The dendrogram on top shows the relationship between the control and the Lmx1a/b cKO samples. We had 3 biological replicates for the control and 4 biological replicates for the Lmx1a/b cKO mutants, with two technical replicates per sample. The map was produced with the function pheatmap in the R package pheatmap, and shows the relationships between the significantly differently expressed genes. **b)** The principle component analysis (PCA) plot shows the relationship between the RNA-seq samples with respect to two axes, which explain 67% and respectively 16% of the variance. The plot indicates a good correlation between the Lmx1a/b cKO samples whereas we see slight differences in the control samples. The Lmx1a/b cKO samples cluster together with respect to PC1 and PC2, while the control samples are scattered with respect to these principal components. However, as seen from the heatmap in **a**, the mutant samples cluster together and so do the control samples. The PCA plot was obtained using the output of DEseq2 function rlog, which minimizes the differences between samples for rows with small counts and with respect to library size. **c)** Scatterplot of log2 fold changes versus the mean of normalised counts. The graph shows unshrunken maximum likelihood estimates. Dots in red are the significantly differentially expressed genes.

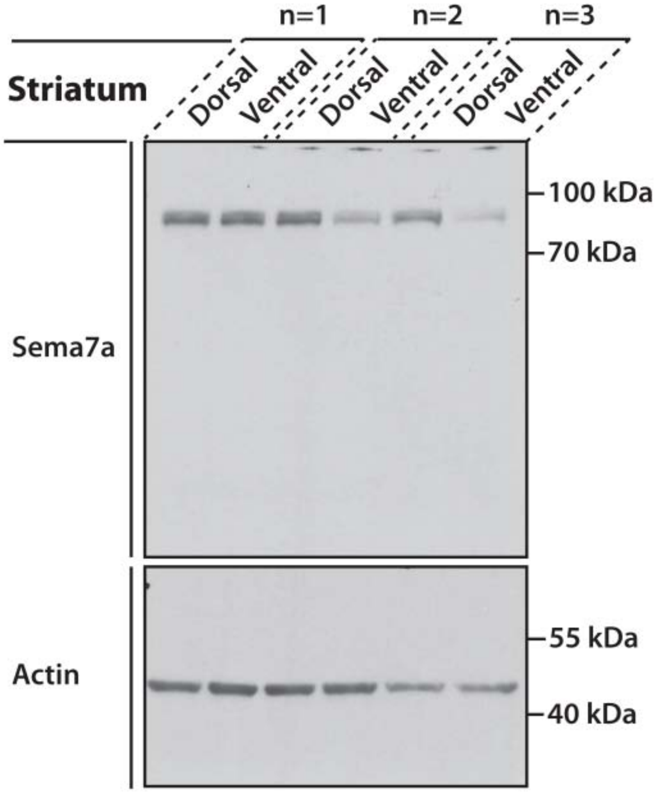

Supplementary Figure 7: Full gel image for the western blot shown in Figure 5 c.

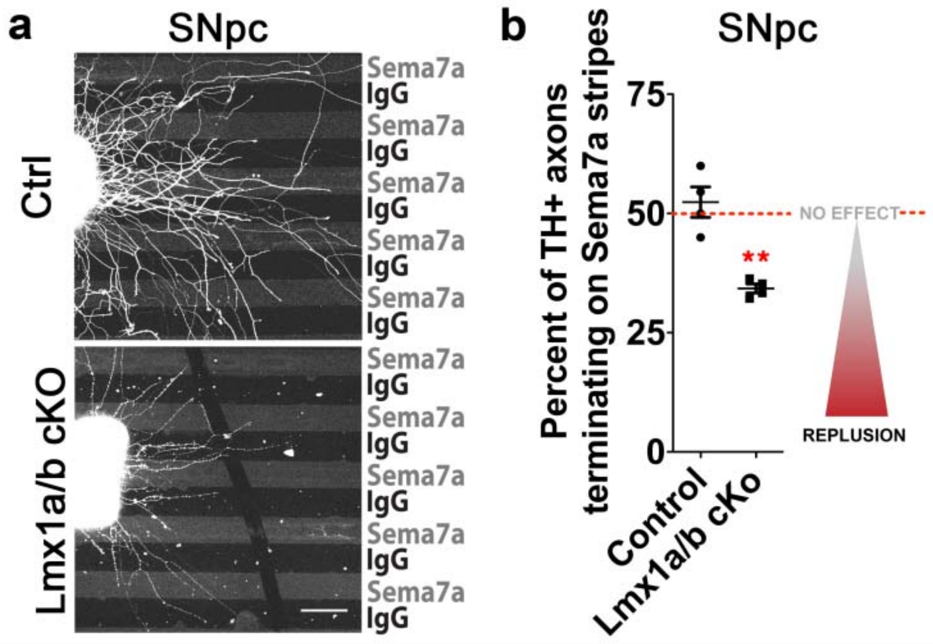

Supplementary Figure 8: SNpc mDA axons from Lmx1a/b cKO embryos are repelled by Sema7a stripes. (a) Confocal images of stripes assay showing explants from SNpc of control (upper panel) and Lmx1a/b cKO embryos (lower panel). (**b**) Quantification of the number of DA axons terminating on Sema7a stripes or on IgG stripes (n=4 independent experiments, Mann-Whitney U, p = 0.0286). Scale bars: 150 μm.

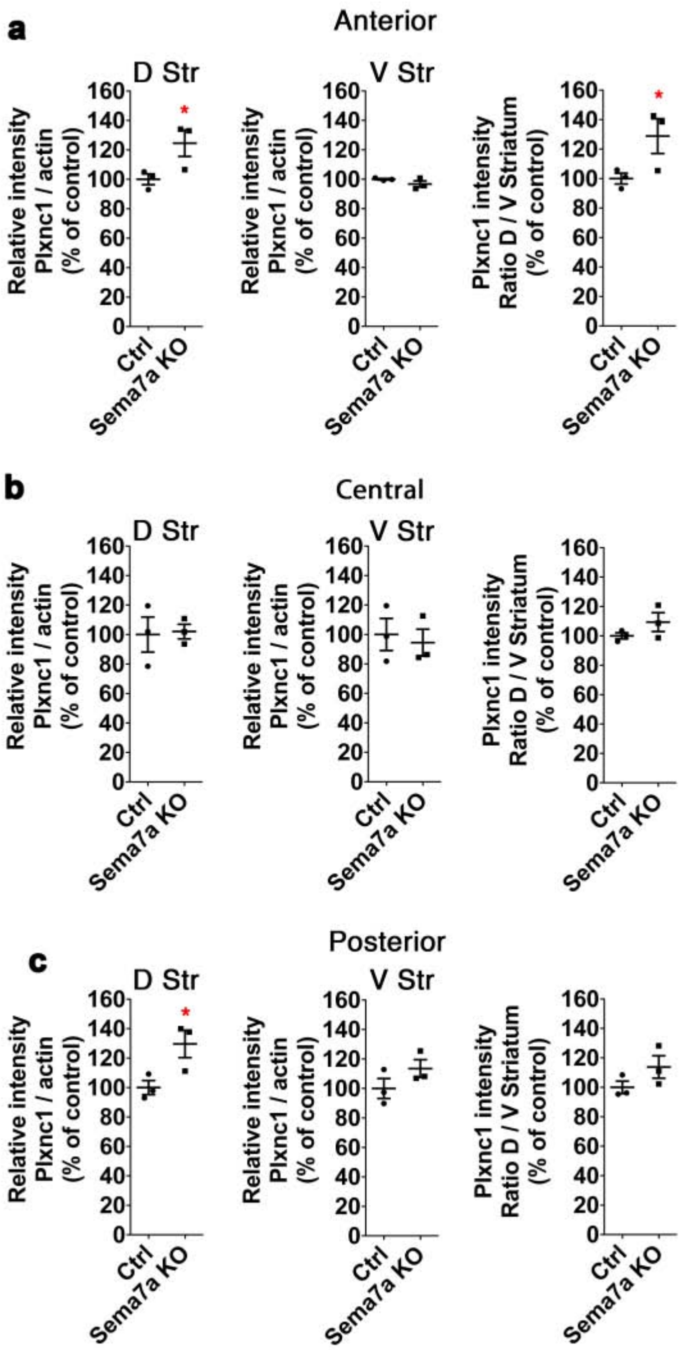

Supplementary Figure 9: Quantification of Plxnc1 axonal density in dorsal and ventral striatal regions of WT and Sema7a KO**.** Graphs in a, b and c show the quantification at anterior, central and posterior striatal regions respectively. (n=3, one-tailed unpaired t-test, $p^{(Ant\ D)}$ = 0.0321, $p^{(Ant\ V)}$ = 0.1060, $p^{(Cent\ D)}$ = 0.4404, $p^{(Cent\ V)}$ = 0.3586, $p^{(Post\ D)}$ = 0.0232, $p^{(Post\ V)}$ = 0.1046, $p^{(Ant\ D/V)}$ = 0.0399, $p^{(Cent\ D/V)}$ = 0.1179, $p^{(Post\ D/V)}$ = 0.0941).

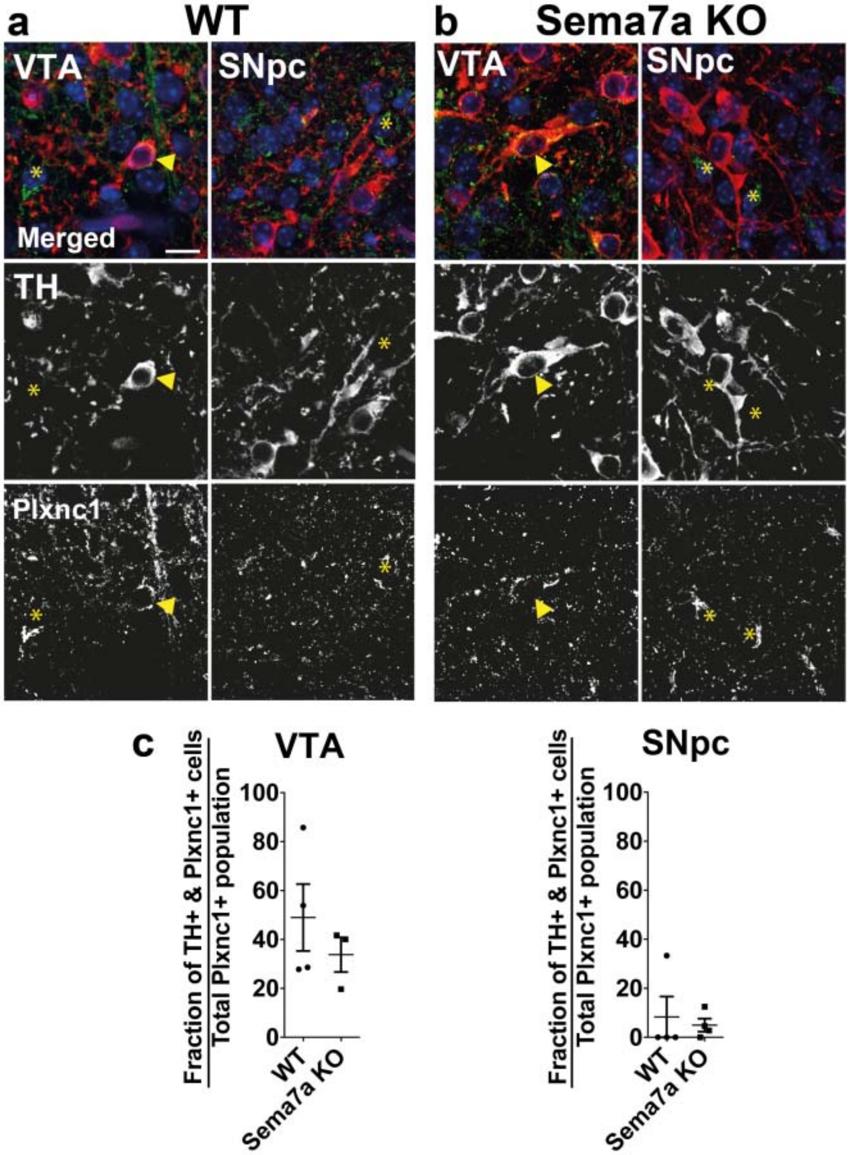

Supplementary Figure 10: Plxnc1 levels are not altered in the SNpc or VTA of Sema7A KO mice. (a-b) Confocal images of Plxnc1 and TH immunostaining in coronal midbrain sections of WT and Sema7a KO mice. Plxnc1 staining barely localizes with TH staining in the SNpc in Sema7A WT and KO animals (asterisk). In the VTA co-localization between Plxnc1 and TH is more common (yellow arrow). Scale bar is 10μm. (**c**) Quantification of the fraction Plxnc1+ cells also expressing TH+ is shown for the VTA and SN in Sema7A WT and KO mice. No difference in the amount of double positive cells were found (4 sections from two animals, two-tailed unpaired t-test: $p^{(VTA)} = 0.418$, $p^{(SNpc)} = 0.715$).

Supplementary Table 1. Differentially expressed genes between Lmx1a/b cKO and control identified by RNA sequencing from laser capture micro-disected midbrain dopamine region at E15.5. Abbreviations: baseMean: the base mean over all rows, lfcSE: Log Fold Standard Error, stat: Wald statistic, pvalue: Wald test p-value, padj: Benjamini-Hochberg (BH) adjusted p-values.

Gene symbol	baseMean	log2Fold Change	lfcSE	stat	pvalue	padj
Tec	132.63489	1.7691725	0.3127767	5.6563444	1.55E-08	0.0001223
Kank4	648.28329	1.6044785	0.2788192	5.7545482	8.69E-09	0.0001223
Gxylt2	117.22443	1.5751485	0.3160579	4.9837336	6.24E-07	0.0013313
Mroh7	119.47238	1.5390824	0.3083197	4.9918395	5.98E-07	0.0013313
Jade2	135.62344	1.5075358	0.3080761	4.8933875	9.91E-07	0.0016111
Gm26697	108.43484	1.5008836	0.3074238	4.8821323	1.05E-06	0.0016111
Fam83d	104.09539	1.4250111	0.3053186	4.6672922	3.05E-06	0.0037123
Pls1	116.17728	1.336951	0.3023687	4.421592	9.80E-06	0.0103287
Dusp27	88.542255	1.2799397	0.3004672	4.2598312	2.05E-05	0.0179725
	680.38792	1.2758896	0.233943	5.4538485	4.93E-08	0.0002598
Slc38a4	122.74667	1.2447294	0.2988226	4.1654455	3.11E-05	0.0258622
Nmrk1	88.305915	1.2396087	0.2988412	4.148052	3.35E-05	0.0265118
Nsun7	499.66734	1.2243704	0.2830788	4.3251925	1.52E-05	0.0141758
Klhl3	286.16943	1.2144639	0.3040753	3.9939581	6.50E-05	0.0356607
Spata45	79.635073	1.2049569	0.2974424	4.0510598	5.10E-05	0.0315814
Gm13563	78.827415	1.2025212	0.3040513	3.9549938	7.65E-05	0.0390411
Dcaf12l2	66.998171	1.161501	0.2956036	3.9292518	8.52E-05	0.0421073
Plvap	94.530995	1.0947537	0.2972602	3.6828127	0.0002307	0.0703543
Slc7a2	67.063699	1.0887315	0.2918965	3.7298547	0.0001916	0.0688549
Gm26759	64.540308	1.076117	0.2912438	3.6949013	0.00022	0.0703543
Slc25a54	101.58176	1.062969	0.2901577	3.6634178	0.0002489	0.0703543
Foxl2os	78.250002	1.0510832	0.2896624	3.628649	0.0002849	0.0758937
Chrnb3	6008.0655	1.0471264	0.2721203	3.8480283	0.0001191	0.0537969
Slc6a3	28823.34	1.0385222	0.2904993	3.5749566	0.0003503	0.0814569
Lrrc10b	88.673347	1.0353744	0.2886568	3.5868702	0.0003347	0.0801841
Col11a1	4465.2647	1.0344368	0.2823915	3.6631301	0.0002492	0.0703543
Otor	64.590052	1.018518	0.2878937	3.5378262	0.0004034	0.0898525
Gm12504	77.068838	1.0181349	0.3058569	3.3287943	0.0008722	0.1231434
Cubn	74.348658	1.0067295	0.2877886	3.498156	0.0004685	0.0988909
Gm10340	58.21414	1.004274	0.2871122	3.4978453	0.000469	0.0988909
Katnal2	466.15254	1.003239	0.3060688	3.2778216	0.0010461	0.1331852
Irak4	217.69105	0.9969763	0.2873165	3.4699583	0.0005205	0.1028911
Dgka	1936.8967	0.9782067	0.2008982	4.8691661	1.12E-06	0.0016111
Gm14830	68.297434	0.9770787	0.2931843	3.3326428	0.0008603	0.1231434

Sgsh	86.299609	0.9756349	0.2849641	3.4237113	0.0006177	0.107911
Csf2rb2	141.73976	0.9754252	0.2846984	3.4261708	0.0006122	0.107911
Dsc2	70.851927	0.9746244	0.2850363	3.4192995	0.0006278	0.107911
Apobec2	79.340696	0.9743161	0.2849364	3.4194164	0.0006276	0.107911
Slc19a3	60.934841	0.9737366	0.2851052	3.4153589	0.000637	0.1083072
Ulk3	1392.9883	0.9694674	0.3025048	3.2048003	0.0013516	0.1444071
Rec8	66.644822	0.9657843	0.2845039	3.3946257	0.0006872	0.1143904
Map2k1	2399.5915	0.9656409	0.2520367	3.8313509	0.0001274	0.053914
1700001C19Rik	63.791484	0.9648832	0.2854211	3.38056	0.0007234	0.1191547
Pus7	1076.7408	0.9585332	0.2859686	3.3518826	0.0008026	0.1231434
Slc12a7	1860.8765	0.9545708	0.2390951	3.9924307	6.54E-05	0.0356607
Eif2ak2	54.959731	0.9530543	0.2838164	3.3579958	0.0007851	0.1217133
Serpine1	148.70353	0.9473531	0.294872	3.212761	0.0013147	0.1431303
Gm14966	64.669717	0.939529	0.2827349	3.3230029	0.0008905	0.1231434
Slc43a1	63.916405	0.9391906	0.2827214	3.3219646	0.0008939	0.1231434
Gatsl3	74.656523	0.9288374	0.2818733	3.2952303	0.0009834	0.1331852
Ankdd1a	57.406495	0.9279085	0.2820173	3.2902539	0.001001	0.1331852
Cfap45	52.008411	0.9242172	0.2818455	3.279163	0.0010412	0.1331852
Slc6a20a	78.811181	0.9191505	0.2811447	3.2693151	0.0010781	0.1331852
Fgf10	97.224732	0.9156243	0.2812816	3.2551875	0.0011332	0.1367855
3300002I08Rik	75.486992	0.909309	0.2804578	3.2422313	0.001186	0.1374793
Nrg4	57.192668	0.9082436	0.280593	3.2368722	0.0012085	0.1374793
Hace1	1396.2043	0.9056106	0.3013946	3.0047346	0.0026581	0.1913704
Cntnap4	4563.081	0.901953	0.1804086	4.9995007	5.75E-07	0.0013313
Gm29443	86.114214	0.8989346	0.2796083	3.2149781	0.0013045	0.1431303
Gm15834	51.699951	0.8975923	0.2798937	3.2069044	0.0013417	0.1443303
Adamts4	58.464507	0.8946289	0.2795561	3.2001768	0.0013734	0.1447873
C230014O12Rik	672.44857	0.8927198	0.2946038	3.0302382	0.0024436	0.1884917
Fbln1	887.92113	0.8888746	0.2471092	3.5970924	0.0003218	0.0801841
Necab1	1946.1597	0.8850782	0.2361742	3.7475658	0.0001786	0.065664
Htr5a	765.2541	0.884484	0.2628474	3.3650094	0.0007654	0.1217133
1700047F07Rik	46.416472	0.8742544	0.2792779	3.1304104	0.0017456	0.1577344
Gm3411	40.804494	0.8711275	0.2874008	3.0310545	0.002437	0.1884917
Oit1	68.536755	0.8691295	0.2774688	3.1323506	0.0017341	0.1575962
Agfg2	1570.7873	0.8673752	0.2131904	4.0685464	4.73E-05	0.0315814
Gm2897	46.016702	0.8615516	0.2869817	3.0021137	0.0026811	0.1913704
Prpf39	2756.7653	0.856514	0.2072244	4.1332683	3.58E-05	0.0269304
Irgm1	66.55605	0.8433621	0.2754112	3.0621931	0.0021972	0.1814455
AF529169	1622.9676	0.8428367	0.2440478	3.4535722	0.0005532	0.1053973

Adgrg3	57.070368	0.8419657	0.2754121	3.0571123	0.0022348	0.1814455
Med12	4334.9881	0.8416678	0.2667436	3.1553446	0.0016031	0.1501429
Zfp937	77.945504	0.8330366	0.2744545	3.0352445	0.0024034	0.1884917
Gm23455	46.155624	0.8317904	0.2747444	3.0275064	0.0024658	0.1888374
Rasip1	1855.5569	0.8270402	0.1858381	4.4503265	8.57E-06	0.0096843
Phldb2	1442.6985	0.8257672	0.2643365	3.1239239	0.0017846	0.1603372
Klf10	608.40906	0.8245077	0.2475375	3.3308394	0.0008658	0.1231434
Pigl	585.68591	0.8173411	0.2489093	3.2836909	0.0010246	0.1331852
Atg4c	758.67684	0.8059936	0.2483192	3.2457963	0.0011712	0.1374793
Pakap	3089.6065	0.8055926	0.2639465	3.0521058	0.0022724	0.1824049
Trpm7	3483.4636	0.8009433	0.252056	3.1776405	0.0014848	0.1464889
Ntsr1	1769.0789	0.7682348	0.2231693	3.4423862	0.0005766	0.1060218
9330182L06Rik	2540.2536	0.7591052	0.239802	3.1655496	0.0015479	0.1478222
Mdn1	5277.8838	0.7495314	0.2290041	3.2730043	0.0010641	0.1331852
Nbeal2	2439.9825	0.7247411	0.2081758	3.4813907	0.0004988	0.1024389
Tnfrsf19	2651.0312	0.7079271	0.1902176	3.7216702	0.0001979	0.0690217
Abcg4	5679.8077	0.6941309	0.1914374	3.6258907	0.000288	0.0758937
Gm15952	1098.9068	0.6722274	0.1943233	3.4593248	0.0005415	0.1044297
Gm45837	2147.9732	0.6678855	0.2146904	3.1109238	0.001865	0.1666199
Htt	1771.5858	0.653338	0.1767462	3.6964754	0.0002186	0.0703543
Xpo1	8722.7451	0.650828	0.2037043	3.1949646	0.0013985	0.1450659
Pdzrn4	5923.5491	0.6412071	0.2122079	3.0215991	0.0025144	0.1889355
Pcgf6	4844.3849	0.6255757	0.143456	4.3607501	1.30E-05	0.0128102
**Plxnc1**	8780.4919	0.6254371	0.1802095	3.4706121	0.0005193	0.1028911
Gna13	2681.98	0.6111642	0.1894431	3.226109	0.0012549	0.1417359
Gpd2	2977.2057	0.5904261	0.1715085	3.4425476	0.0005763	0.1060218
Kcnn2	2435.1412	0.5467289	0.1808873	3.0224832	0.0025071	0.1889355
Chst1	8781.1933	0.5416485	0.1649311	3.2840906	0.0010231	0.1331852
Cdh4	12776.909	0.5202046	0.1468081	3.5434325	0.000395	0.0892202
Pfkp	57113.287	0.5063947	0.1586659	3.1915785	0.001415	0.1452924
Slc9a3r2	4692.6263	0.5046352	0.1249382	4.0390793	5.37E-05	0.0315814
Bicdl1	11749.212	0.4973351	0.130162	3.8208927	0.000133	0.053914
Mef2d	4361.6344	0.4937621	0.1599727	3.0865405	0.002025	0.1730885
Syn1	18533.855	0.4798082	0.1462833	3.2799925	0.0010381	0.1331852
Xbp1	6605.8528	0.4534775	0.1426002	3.1800625	0.0014724	0.1464889
Plekha5	20895.762	0.4436007	0.1449669	3.0600136	0.0022133	0.1814455
Ano6	6652.3748	0.4407902	0.146064	3.0177874	0.0025463	0.1899257
Sos1	4905.0622	0.4137507	0.1275603	3.2435706	0.0011804	0.1374793
Iqsec3	8639.7475	0.4081129	0.1335791	3.0552149	0.002249	0.1814455

Arhgap39	9568.5596	0.3691208	0.1219528	3.0267509	0.002472	0.1888374
Lingo1	15684.562	0.3672232	0.1089274	3.3712645	0.0007482	0.1209287
Wdr82	22052.507	-0.231293	0.0770595	-3.001485	0.0026867	0.1913704
Cdc16	11971.295	-0.274198	0.0796512	-3.442483	0.0005764	0.1060218
Stip1	16039.935	-0.343856	0.1085787	-3.166879	0.0015408	0.1478222
Slc30a9	10966.921	-0.361795	0.105251	-3.437451	0.0005872	0.1067316
Klhdc2	13104.46	-0.362343	0.0886787	-4.086023	4.39E-05	0.0315419
Arfip2	9436.2949	-0.36931	0.1032279	-3.577616	0.0003467	0.0814569
Pmpcb	5703.8052	-0.376154	0.1218673	-3.086584	0.0020247	0.1730885
Stoml1	9440.9333	-0.37891	0.1140804	-3.321434	0.0008956	0.1231434
Hsp90aa1	17776.487	-0.42089	0.1405934	-2.993669	0.0027565	0.1954609
Ldhb	14923.915	-0.434539	0.140344	-3.096244	0.0019599	0.1712668
Fkbp4	31362.249	-0.437285	0.145586	-3.003616	0.0026679	0.1913704
Elp5	10174.683	-0.451532	0.1403834	-3.216422	0.001298	0.1431303
Ranbp1	16985.812	-0.483263	0.1523219	-3.172644	0.0015106	0.1474491
Aamp	10485.908	-0.484909	0.1560144	-3.108106	0.0018829	0.1672721
Dnajc8	13813.461	-0.485091	0.1571407	-3.086988	0.002022	0.1730885
Ubxn6	9882.8166	-0.487475	0.1456868	-3.346048	0.0008197	0.1231434
Msantd3	2305.7181	-0.488881	0.1503843	-3.25088	0.0011505	0.1374793
Gpx4	5278.8232	-0.489146	0.1556935	-3.141725	0.0016796	0.1544117
Map3k7	6738.7591	-0.490067	0.1363892	-3.59315	0.0003267	0.0801841
Atp5d	15609.958	-0.514091	0.1682309	-3.055864	0.0022441	0.1814455
Enpp2	2759.819	-0.523814	0.1734022	-3.020803	0.0025211	0.1889355
Gpat4	13488.82	-0.524783	0.1652165	-3.176337	0.0014915	0.1464889
1700025G04Rik	3412.5692	-0.528299	0.1586236	-3.33052	0.0008668	0.1231434
Trak2	3620.9587	-0.534041	0.1724843	-3.096172	0.0019604	0.1712668
Zbtb8b	2497.156	-0.534141	0.1584601	-3.37082	0.0007494	0.1209287
Pycr2	3781.4407	-0.537003	0.1639172	-3.276062	0.0010527	0.1331852
Rgs2	7293.9668	-0.542005	0.1802151	-3.007547	0.0026337	0.1913704
Phax	9629.9696	-0.545997	0.1816663	-3.005496	0.0026515	0.1913704
Ccnh	4711.3792	-0.546421	0.1423826	-3.837699	0.0001242	0.053914
Tbl3	4946.6772	-0.546734	0.1796033	-3.044118	0.0023336	0.1854362
Cdc34	7285.2973	-0.547387	0.138286	-3.958365	7.55E-05	0.0390411
Nubp2	3904.3301	-0.563735	0.1772675	-3.180138	0.001472	0.1464889
Gm6485	9866.6399	-0.568901	0.1546199	-3.679352	0.0002338	0.0703543
Tpgs1	5931.4605	-0.583849	0.1665095	-3.506401	0.0004542	0.0983894
Mkks	1463.1873	-0.585822	0.1631668	-3.590326	0.0003303	0.0801841
Ttc9b	5257.3612	-0.585825	0.1856507	-3.155524	0.0016021	0.1501429
Coprs	6198.7942	-0.609322	0.201665	-3.021456	0.0025156	0.1889355

Gm12396	2177.1325	-0.619672	0.1922448	-3.223351	0.001267	0.1420929
Tmem120a	4570.7877	-0.625684	0.2077577	-3.011605	0.0025987	0.1913704
Zmat5	1332.6763	-0.628298	0.1921183	-3.27037	0.0010741	0.1331852
Fez2	2739.019	-0.629224	0.195799	-3.21362	0.0013107	0.1431303
Mgst3	6982.0183	-0.631221	0.2074869	-3.04222	0.0023484	0.1856762
Foxo3	3028.9986	-0.660803	0.2146216	-3.078921	0.0020775	0.1756779
Polr2e	8290.4618	-0.672718	0.2075794	-3.240776	0.001192	0.1374793
2010107E04Rik	7787.1436	-0.679603	0.2030118	-3.347604	0.0008151	0.1231434
Uqcr10	7751.0754	-0.681152	0.2281915	-2.985	0.0028358	0.199299
Rplp0	54659.937	-0.684637	0.2131984	-3.211267	0.0013215	0.1431303
Mul1	2376.2704	-0.685379	0.2109351	-3.249241	0.0011571	0.1374793
2410015M20Rik	4596.6937	-0.695344	0.2184111	-3.183647	0.0014543	0.1464889
Dpf3	1994.5975	-0.695847	0.2035029	-3.419347	0.0006277	0.107911
Rnf213	550.34675	-0.698045	0.21896	-3.188001	0.0014326	0.146153
Nedd8	16238.713	-0.700818	0.2311376	-3.032036	0.0024291	0.1884917
Srp9	4614.9831	-0.70677	0.21182	-3.336653	0.0008479	0.1231434
Gm10221	10624.915	-0.709061	0.1837048	-3.859783	0.0001135	0.0527818
Gm12350	3700.0572	-0.744447	0.2470567	-3.013264	0.0025845	0.1913704
Timm8b	8773.3091	-0.747128	0.2336557	-3.197559	0.001386	0.1450659
Rpl34	5556.2326	-0.752002	0.250153	-3.006168	0.0026456	0.1913704
Tmem178	1636.4353	-0.756022	0.2250024	-3.360061	0.0007793	0.1217133
Ctdsp1	1139.025	-0.756189	0.2440974	-3.097897	0.001949	0.1712668
Fis1	23677.435	-0.775178	0.2523253	-3.07214	0.0021253	0.1768811
Tmem256	5462.202	-0.777676	0.2543954	-3.056956	0.002236	0.1814455
Fxn	1057.1254	-0.780662	0.2221675	-3.513843	0.0004417	0.0970026
Nlrc4	3124.3561	-0.780821	0.2573248	-3.034381	0.0024103	0.1884917
Hist3h2ba	13657.2	-0.785568	0.2071507	-3.792255	0.0001493	0.0562061
Mrpl41	6742.02	-0.785757	0.2400234	-3.273669	0.0010616	0.1331852
Rpl19	4207.8326	-0.791169	0.2499888	-3.16482	0.0015518	0.1478222
Itgb1bp1	1798.4161	-0.800114	0.2535967	-3.155062	0.0016046	0.1501429
Rnf7	7999.9667	-0.80564	0.2108462	-3.820983	0.0001329	0.053914
Gm1673	17436.238	-0.814416	0.2706502	-3.009108	0.0026202	0.1913704
Psmb4	18137.743	-0.822282	0.2620043	-3.138429	0.0016986	0.1552565
Alkbh4	1539.8707	-0.836233	0.2276993	-3.672534	0.0002402	0.0703543
Rpl13a	54654.197	-0.843804	0.2666012	-3.165041	0.0015506	0.1478222
Znrd1	3029.2497	-0.849079	0.1649199	-5.148429	2.63E-07	0.0010384
Fhl2	1065.4021	-0.857417	0.2570051	-3.336189	0.0008494	0.1231434
2310009A05Rik	753.18698	-0.857582	0.2475742	-3.463941	0.0005323	0.1039215

Uqcr11	8221.8771	-0.865363	0.2650204	-3.265268	0.0010936	0.1340555
Ust	452.53199	-0.883043	0.2680937	-3.293785	0.0009885	0.1331852
Tacr1	931.62202	-0.886526	0.2195494	-4.037935	5.39E-05	0.0315814
Fxyd2	516.49703	-0.893564	0.2760348	-3.237142	0.0012073	0.1374793
Gm10073	10897.628	-0.900213	0.2928964	-3.073485	0.0021157	0.1768811
Gm8186	7436.661	-0.910486	0.2943147	-3.093579	0.0019776	0.171821
Fmc1	3660.2474	-0.910743	0.2396009	-3.801082	0.0001441	0.0562061
Scand1	5486.2763	-0.912363	0.2675907	-3.409546	0.0006507	0.109465
Sdhaf1	921.41393	-0.915346	0.2978267	-3.073417	0.0021162	0.1768811
Cnp	2088.7242	-0.921781	0.1919947	-4.801078	1.58E-06	0.0020796
BC026585	338.98832	-0.942816	0.3156686	-2.986727	0.0028198	0.1990611
Gm5617	523.26568	-0.949182	0.3115639	-3.046508	0.0023152	0.1848973
Igfbpl1	2184.4764	-0.953839	0.3093933	-3.082934	0.0020497	0.1742583
Rps14	32336.088	-0.964627	0.2474953	-3.897556	9.72E-05	0.0465612
Med18	591.73258	-0.973819	0.264042	-3.688123	0.0002259	0.0703543
Snrpe	5563.4135	-0.97818	0.2712029	-3.606819	0.00031	0.0801841
Boll	2287.175	-0.981266	0.3065337	-3.201167	0.0013687	0.1447873
H19	12356.601	-0.982634	0.1977588	-4.968848	6.74E-07	0.0013313
Rpl5-ps2	76.775066	-0.984702	0.2951819	-3.335917	0.0008502	0.1231434
Cpa2	307.02543	-0.986434	0.3136498	-3.145016	0.0016608	0.1538591
Rpl9	2126.3451	-0.986685	0.2780452	-3.548651	0.0003872	0.0887385
Mdfi	320.78235	-0.987827	0.3107094	-3.179264	0.0014765	0.1464889
Tbc1d4	359.55886	-0.990889	0.3102433	-3.19391	0.0014036	0.1450659
Zfp335os	661.8285	-0.993406	0.2724853	-3.645724	0.0002666	0.0739715
Gm15286	212.83742	-0.994512	0.316272	-3.144481	0.0016638	0.1538591
Gm2199	611.87128	-1.002211	0.2870517	-3.491394	0.0004805	0.099977
Agt	108.33311	-1.005079	0.3085674	-3.257242	0.001125	0.1367855
Siva1	1284.0794	-1.029746	0.2778454	-3.706182	0.0002104	0.0703543
Zfp36l1	637.50493	-1.065304	0.3168151	-3.362541	0.0007723	0.1217133
Cd160	289.75252	-1.075188	0.3091582	-3.477792	0.0005056	0.1024931
Nol3	289.97327	-1.130848	0.3110404	-3.635694	0.0002772	0.0755843
Gm4876	150.26848	-1.137648	0.3169924	-3.588881	0.0003321	0.0801841
Chst7	485.80818	-1.159365	0.3160115	-3.668744	0.0002437	0.0703543
Gm9748	220.55359	-1.178057	0.31685	-3.718028	0.0002008	0.0690217
4933431K14Rik	248.92463	-1.203263	0.3168666	-3.797381	0.0001462	0.0562061
Loxl1	246.74786	-1.281964	0.3170186	-4.043814	5.26E-05	0.0315814
